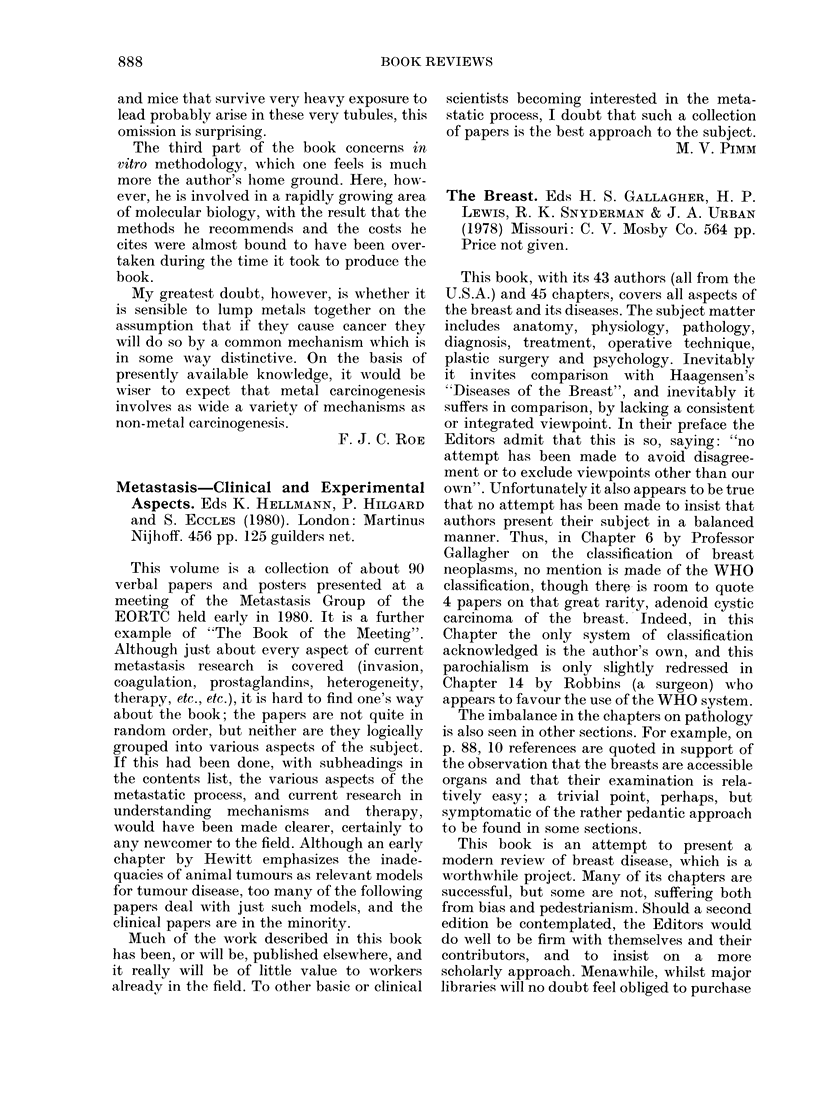# Metastasis—Clinical and Experimental Aspects

**Published:** 1981-06

**Authors:** M. V. Pimm


**Metastasis—Clinical and Experimental Aspects.** Eds K. Hellmann, P. Hilgard and S. Eccles (1980). London: Martinus Nijhoff. 456 pp. 125 guilders net.

This volume is a collection of about 90 verbal papers and posters presented at a meeting of the Metastasis Group of the EORTC held early in 1980. It is a further example of "The Book of the Meeting". Although just about every aspect of current metastasis research is covered (invasion, coagulation, prostaglandins, heterogeneity, therapy, *etc*., *etc*.), it is hard to find one's way about the book; the papers are not quite in random order, but neither are they logically grouped into various aspects of the subject. If this had been done, with subheadings in the contents list, the various aspects of the metastatic process, and current research in understanding mechanisms and therapy, would have been made clearer, certainly to any newcomer to the field. Although an early chapter by Hewitt emphasizes the inadequacies of animal tumours as relevant models for tumour disease, too many of the following papers deal with just such models, and the clinical papers are in the minority.

Much of the work described in this book has been, or will be, published elsewhere, and it really will be of little value to workers already in the field. To other basic or clinical scientists becoming interested in the metastatic process, I doubt that such a collection of papers is the best approach to the subject.

M. V. Pimm